# Genome assemblies for *Chromidotilapia guntheri* (Teleostei: Cichlidae) identify a novel candidate gene for vertebrate sex determination, RIN3

**DOI:** 10.3389/fgene.2024.1447628

**Published:** 2024-08-16

**Authors:** Kristen A. Behrens, Stephan Koblmüller, Thomas D. Kocher

**Affiliations:** ^1^ Department of Biology, University of Maryland, College Park, MD, United States; ^2^ Institute of Biology, University of Graz, Graz, Austria

**Keywords:** sex chromosome, sex determination, African cichlids, genome assembly, RIN3

## Abstract

Advances in genome sequencing have greatly accelerated the identification of sex chromosomes in a variety of species. Many of these species have experienced structural rearrangements that reduce recombination between the sex chromosomes, allowing the accumulation of sequence differences over many megabases. Identification of the genes that are responsible for sex determination within these sometimes large regions has proved difficult. Here, we identify an XY sex chromosome system on LG19 in the West African cichlid fish *Chromidotilapia guntheri* in which the region of differentiation extends over less than 400 kb. We develop high-quality male and female genome assemblies for this species, which confirm the absence of structural variants, and which facilitate the annotation of genes in the region. The peak of differentiation lies within *rin3*, which has experienced several debilitating mutations on the Y chromosome. We suggest two hypotheses about how these mutations might disrupt endocytosis, leading to Mendelian effects on sexual development.

## 1 Introduction

Three classes of genes are frequently identified in studies of sex-determination in teleosts (Kitano et al., 2024). The first class of genes are elements of the TGFß pathway, which transduces extracellular signals to the nucleus. A duplication of the ligand *amh* is a sex determiner in Nile tilapia (*Oreochromis niloticus*) (Li et al., 2015) and Northern pike (*Esox lucius*) (Pan et al., 2019). Variations of the amh receptor are implicated in sex determination in several orders, including Cichliformes, Siluriformes, and Tetraodontiformes (Kamiya et al., 2012; Nacif et al., 2023; Wen et al., 2023). Polymorphisms of the ligand *gsdf* are associated with sex determination in the Philippine medaka (*Oryzias luzonensis*) (Myosho et al., 2012) and in cichlids from the Malawi basin (Munby et al., 2021). A recent study associated polymorphisms in bone morphogenetic protein receptor type 1b (*bmprt1*) with sex determination in Atlantic herring (*Clupea harengus*) (Rafati et al., 2020). The second class of genes are involved in steroid metabolism. Variation in steroid modification enzymes contribute to sex determination, including *hsd17b1* in amberjacks (*Seriola* spp.) (Koyama et al., 2019) and *sult1st6y* in bluefin tunas (*Thunnus maccoyii* and *T. orientalis*) (Nakamura et al., 2021). The third class of genes include transcription factors such as *dmrt* and *sox. Dmrt* has taken on a role in sex determination in the Japanese ricefish (*Oryzia curvinotus* and *O. latipes*) (Matsuda et al., 2003) and a flatfish (*Cynoglossus semilaevis*) (Chen et al., 2014). *Sox2* is implicated in sex determination in another flatfish (*Scophthalmus maximus*) (Martínez et al., 2021) and *sox3* is associated with sex in the Indian ricefish (*Oryzias dancena*) (Takehana et al., 2014).

While most Mendelian factors associated with sex determination are part of these well-studied pathways, there are exceptions. A surprising role for an immune gene (*irf9*/*sdY*) was identified in salmonids (Yano et al., 2012; Yano et al., 2013). In *Solea senegalensis*, a species of flatfish, an allele of the receptor of the follicle stimulating hormone (*fshr*) inhibits the action of the follicle stimulating hormone tipping the gonad into testis (de la Herrán et al., 2023). Finally, in the Atlantic cod (*Gadus morhua*), a copy of the zinc knuckle gene (*zkY*) on the Y chromosomes leads to male development (Kirubakaran et al., 2019). So, while some genes have been evolved to become Mendelian factors in sex determination convergently in multiple lineages, this does not preclude the evolution of sex-determiners from other types of genes. Sex-determiners have been mapped in many other species, but the sex-linked regions do not appear to contain candidate genes in any of the three gene classes already identified. The true number of genes capable of becoming sex determiners is still unknown, and it seems likely that more genes will be implicated as we investigate sex determination in more species (Tao et al., 2021b; Behrens et al., 2024a).

There are multiple methodologies for identifying sex chromosomes, the effectiveness of which are dependent on properties of the sex chromosomes themselves (Palmer et al., 2019). Highly divergent sex chromosomes such as those of mammals (Hughes and Page, 2015) or birds (Zhou et al., 2014) are often visibly different when karyotyped and can be detected by looking for differences in DNA sequence coverage between the sexes (Vicoso et al., 2013; Darolti et al., 2019). Detection of homomorphic sex chromosomes, which are not detectable by karyotyping, is much more challenging. Many fish, including cichlids, have homomorphic sex chromosomes (Kitano and Peichel, 2012), which can be identified by examining the distribution of sex-specific SNPs along the chromosomes (Gammerdinger et al., 2018; Behrens et al., 2024a).

Characterization of the sex-determining genes on these sex chromosomes is critical to understanding the developmental mechanisms of sex determination. However, identification of a causative gene or variant within the sex-determining region has been challenging, especially when they are contained within structural variants that sequester differentiation across large genomic regions encompassing dozens to hundreds of genes. An example of this is the discovery of the Y-linked *figla-like* gene, proposed to have a role in sex determination in Nile tilapia, that is not present in the current assembly of an XX *O*. *niloticus* genome, but which was identified in an assembly of the *Oreochromis aureus* genome (Curzon et al., 2022). Another challenge in identifying candidate genes is the distance of the closest quality reference genome from the organism of interest (Darolti et al., 2022). Distantly related species may have accumulated structural differences, such as inversions, gene insertions, or gene duplications that can obscure the gene content of the sex-determining region.

Sex chromosomes often feature highly repetitive or degenerated regions that are difficult to assemble. To avoid this complication, assemblies were often generated only for the homogametic sex (Rhie et al., 2021; Carey et al., 2022). The advancement and reduction in cost of long read sequencing technologies such as PacBio HiFi and Oxford Nanopore now allow us to assemble sex chromosomes from long reads that can span these repetitive regions. An additional challenge with sex chromosomes is that while assemblers like hifiasm (Cheng et al., 2021) are able to phase autosomes, they may struggle to determine haplotypes in sex chromosomes due to potential changes in structure, gene content, and repeat content (Carey et al., 2022). Despite this, assembly of sex chromosomes is becoming increasingly successful. There were several early attempts to assemble the human Y chromosome from telomere to telomere (Skaletsky et al., 2003; Jain et al., 2018), but a complete assembly was only accomplished recently (Rhie et al., 2023). Relatively complete assemblies of the sex chromosomes have been completed for several fish, including the stickleback (Peichel et al., 2020), the zig-zag eel (Xue et al., 2021), and the spotted knifejaw (Li et al., 2021).

Cichlid fishes (Cichlidae) are a large and incredibly diverse group, featuring upwards of 1,500 species in Africa alone, which makes them an ideal group for studying the mechanisms of speciation, morphological divergence, and other evolutionary processes (Burress, 2015; Santos et al., 2023). They have proven to be a good system in which to study the evolution of sex chromosomes (El Taher et al., 2021). The development of long read sequencing technologies has allowed for several high quality cichlid assemblies, including *O*. *niloticus* and *Metriaclima (Maylandia) zebra* (Conte and Kocher, 2015; Conte et al., 2019), and *O. aureus* and *O*. *mossambicus* (Tao et al., 2021c).

A great diversity of sex determination systems have been characterized in East African cichlids (Gammerdinger and Kocher, 2018; Behrens et al., 2024b). *Chromidotilapia guntheri* is a paternally mouthbrooding West African cichlid. It is a member of the tribe Chromidotilapiini, a species-rich early-branching lineage within the African cichlid tree (Greenwood, 1987; Schwarzer et al., 2015; Astudillo-Clavijo et al., 2023). This places it as an outgroup to all African cichlid species that have previously been investigated for sex chromosomes. The closest relatives of *C. guntheri* that have been studied for sex chromosomes are members of the tribe Oreochromini. *Oreochromis niloticus* has an XY-LG23 system in some strains (Conte et al., 2017), and an XY-LG1 system in a Japanese strain (Tao et al., 2021c). *O. aureus* has a ZW-LG3 system (Tao et al., 2021c), and *O. mossambicus* segregates both XY-LG14 and a ZW-LG3 systems (Tao et al., 2021a). The enormity of the cichlid radiation means genome assemblies for many individual clades may be needed to understand the evolution of sex chromosomes.

Karyotype work shows *C. guntheri* has a diploid chromosomal number of 48 (Ozouf-Costaz et al., 2017). The only other known karyotype for the Chromidotilapiini is *Pelvicachromis pulcher*, a species of cichlid with environmental sex determination driven by temperature and water pH (Reddon and Hurd, 2013; Renn and Hurd, 2021), which also has a diploid chromosomal number of 48 (Post, 1965). This diploid number is greater than the modal number for cichlids, which is 2n = 44 (Feldberg et al., 2003; Poletto et al., 2010), but is close to the ancestral number for teleost fish (2n = 50) (Nakatani et al., 2007). The reduction from 50 to 48 is proposed to result from a fusion of two acrocentric chromosomes, likely forming LG7 (Liu et al., 2013; Ozouf-Costaz et al., 2017; Conte et al., 2019) and creating what was likely the ancestral karyotype for most cichlids (2n = 48) (Poletto et al., 2010). LG23 was also formed by a fusion (Conte et al., 2017; Conte et al., 2019) however this occurred only in the African cichlid lineage.

The goals of this study were to 1) look for evidence of genetic sex determination in *C. guntheri*, 2) map the sex determining loci, and 3) characterize the genes within these regions that might affect sexual development.

## 2 Materials and methods

### 2.1 Pooled-sequencing

The *Chromidotilapia guntheri* used were full siblings reared at the University of Graz from aquarium stock. We sampled tissue from 23 males and 26 females, and individuals were dissected to verify sex by visual inspection of gonad morphology. Animal care and use were approved under animal care protocols BMWFW-66.007/004-WF/V/3b/2016 (University of Graz) and R-OCT-22-46 (University of Maryland) and this study was carried out with the approval of the ethics committee of the University of Graz (permit number GZ. 39/115/63 ex 2022/23). DNA was purified separately for each individual using phenol:chloroform extraction in phase-lock silica gel tubes (Quantabio, Beverly MA, United States). The DNA from each individual was quantified by Picogreen fluorescent assay (ThermoFisher Scientific, Waltham, Massachusetts, United States) and then equimolar pools were constructed for males and for females. Sequencing libraries were constructed for 150bp paired-end DNA sequencing on a NovaSeq6000 S4 (Illumina, San Diego CA, United States) by Maryland Genomics (Institute for Genome Sciences, Baltimore MD, United States).

### 2.2 Sex-specific SNP analysis

The main basis of our analyses is the identification and analysis of sex-specific SNPs. These SNPs were identified following our methods described previously (Behrens et al., 2022) using the sex-SNP-finder pipeline (Gammerdinger et al., 2018). Previously reported code from that study is available (https://github.com/Gammerdinger/sex-SNP-finder). Briefly, the sequence reads were aligned with BWA version 0.7.12 using the default parameters along with read group labels. We initially aligned all samples to the closest high-quality reference assembly, *O*. *niloticus* UMD_NMBU (RefSeq GCF_001858045.2), which was the best assembly available at the time of our initial analysis. At each variable nucleotide site, we calculated the *F*_ST_ statistic between the populations of male and female sequence reads. The resulting *F*_ST_ plots provide a first indication of the differentiation between male and female genomes. We further identified XY- and ZW- patterned SNPs as SNPs that were fixed (frequency less than 0.1) in one sex and polymorphic (frequency between 0.3 and 0.7) in the other sex. Separate plots of the allele frequency of XY- and ZW- patterned SNPs suggested the type of heterogametic system segregating (XY or ZW). This process was repeated against the new *C*. *guntheri* assembly (this study, JBDKXC000000000).

We used Bedtools *make windows* and *coverage* to calculate the density of sex-patterned SNPs in 100kbp windows across the genome. We identified the top 1% of windows (∼78 of 7,800 anchored windows) with the highest number of sex-patterned SNPs using the methodology described in Kocher et al. (2022). The log_2_(XY:ZW) ratio of SNP density was then calculated for each window. A Kruskal–Wallis (KW) test on the ranked data was conducted in R (v.2023.03.0+386) using kruskal.test from the *stats* package to determine if the log ratio differed among chromosomes. If the differences were statistically significant, the Dunn’s test from the *rstatix* R package was conducted post-hoc to determine which chromosomes differed significantly from one another with Benjamini–Hochberg correction for multiple tests. Regions of elevated sex-specific SNP density were visualized in IGV to identify candidate sex determining genes.

### 2.3 Genome sequencing

Additional *C. guntheri* were obtained from the aquarium trade and maintained in the Tropical Aquaculture Facility at the University of Maryland. Animal use was approved under the animal care protocol R-OCT-22-46 (U. Maryland) and all experiments were conducted in accordance with the Guide for Care and Use of Laboratory Animals. High-molecular weight DNA was prepared from a single male and a single female individual, which were sexed by visual inspection of gonad morphology. DNA was extracted from heart tissue using the Circulomics/PacBio Nanobind Tissue RT kit and the short-read elimination >25 kb enrichment kit was used to remove shorter reads from the sample. DNA concentrations were quantified by fluorescence spectroscopy using a Quant-iT PicoGreen assay (ThermoFisher, Waltham MA, United States). At the Maryland Genomics center samples were size selected on a BluePippin pulse-field gel system (Sage Science, Beverly MA, United States), sequencing libraries were constructed and extra-long PacBio HiFi sequencing was conducted on the PacBio Revio machine (Pacific Bioscience, Menlo Park CA, United States).

### 2.4 Genome assembly

Genome assemblies were constructed using hifiasm (Cheng et al., 2021; Wang, 2022) with the default purge_dups setting on. Bandage (Wick et al., 2015) was then used to make supported merges based on contiguity of contigs. Contigs for each sex were then aligned against the *O*. *aureus* (ZZ) assembly (GCF_013358895.1) (Tao et al., 2021c), which was the most complete reference available at the time of this analysis, using D-Genies (Cabanettes and Klopp, 2018) to determine placement of contigs (Supplementary Figure S1). This initial alignment was used to inform preliminary manual merges between contigs, where a string of 50 N were used between two contigs to indicate a gap. When necessary, contigs were reverse complemented with seqtk (https://github.com/lh3/seqtk). After this initial assignment of contigs, the male and female assembly were aligned against each other and visualized in D-Genies to further guide joins and identify false joins. The Telomere Identification Toolkit (Tidk, v0.2.31) (https://github.com/tolkit/telomeric-identifier) was used to detect repeats with a pattern consistent with a telomeric identity. Once the repeat was detected (AACCCT and its reverse complement), this was used to guide correct orientation of contigs when scaffolding. Potential false joins were visually inspected in IGV (Thorvaldsdóttir et al., 2013) to determine if the contig produced by hifiasm was supported by the reads.

To identify the Y chromosome, the male haplotigs, the female contig for LG19, and the male and female PacBio HiFi reads were aligned. These alignments were used to identify regions of elevated heterozygosity in the male that were not present in the female. The haplotig with greatest number of male-specific SNPs in this region was inferred to be the Y chromosome. The X and Y were then compared using ModDotPlot (Sweeten et al., 2024).

### 2.5 Quality assessment

Genome assembly quality was assessed using the Genome Evaluation Pipeline (GEP) (https://git.imp.fu-berlin.de/cmazzoni/GEP), which contains the following programs: GenomeScope2 (https://github.com/tbenavi1/genomescope2.0), meryl (Miller et al., 2008; Koren et al., 2017), merqury (Rhie et al., 2020), BUSCOv5 (Manni et al., 2021), and a modified version of assembly_stats (v0.1.4) (http://doi.org/10.5281/zenodo.3968775). The *O. aureus* assembly (Tao et al., 2021c) was used as a guide for expected chromosome size. Coverage was calculated using samtools depth (Danecek et al., 2021).

### 2.6 Annotation of the sex-determining region

The sex-determining region on the new *C. guntheri* male assembly was annotated by aligning genes from the *O. niloticus* and *O. aureus* assemblies in that region against the new assembly using minimap2 (Li, 2018). Once oriented, we aligned the PacBio reads as well as the pooled sequencing reads for both sexes against the haplotigs identified as the Y. The genes of interest were extracted from the assembly fasta using samtools faidx and the region coordinates. This subset fasta was then aligned and annotated using the *O. aureus* annotation of *rin3* in the EMSEMBL tool genewise (Madeira et al., 2022). The protein sequence called by genewise was then used in a multisequence alignment using ClustalOmega (Sievers et al., 2011; Madeira et al., 2022) with *rin3* from both sexes of *C. guntheri*, and the annotations of *rin3* on NCBI of for the outgroup species *O. aureus*, *Haplochromis (Astatotilapia) burtoni*, *Simochromis diagramma*, and *Neolamprologus brichardi*. This analysis was also conducted for the other genes in the region: *adi1*, *tedc1*, *pth2*, and *dnal1*. If the protein had multiple isoforms for a species, all potential isoforms were included in the multisequence alignment.

## 3 Results

### 3.1 Sex chromosome discovery

**FIGURE 1:**
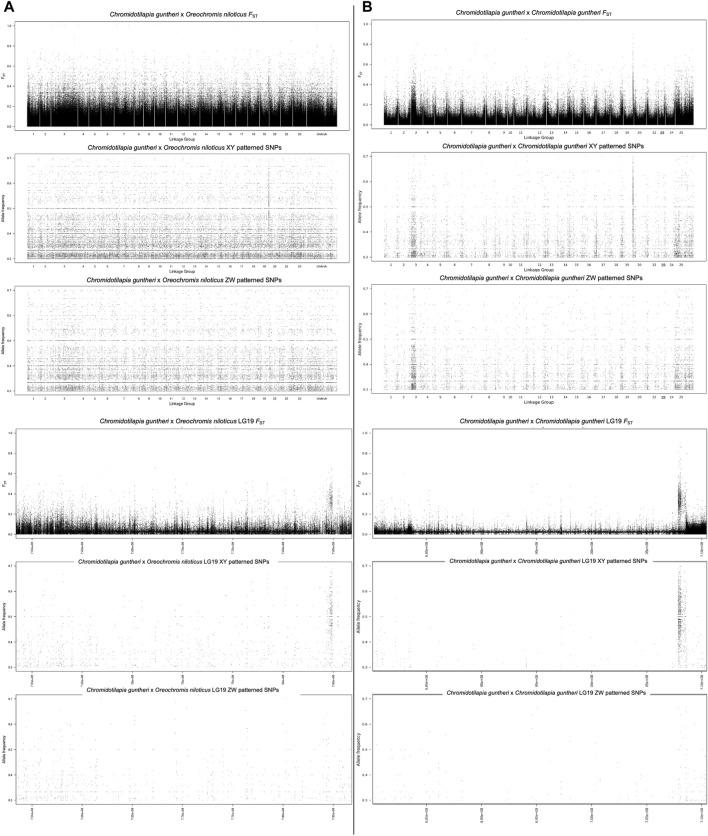
**(A)** Alignment of pooled-seq reads against *O. niloticus* **(B)** alignment of pooled-seq reads against *C. guntheri*.

The analysis of sex-specific SNPs using the *O. niloticus* genome as a reference identified signal on LG19 in *C. guntheri* (Figure 1A). When the statistical methods were conducted based on this alignment, LG3 showed significant signal in the Dunn’s test. In the top 1% analysis, LG19 had 7 XY windows and 2 ZW windows and the top 100 kb window (124 XY SNPs) was on LG19 (Supplementary Table S1). The highest non-LG19 window was on LG1 with 59 XY SNPs. LG3 had 6 XY windows and 7 ZW windows, and the highest window contained 35 XY SNPs. The narrow signal on LG19, which against *O. niloticus* spans approximately 400 kb, was small enough that identifying a candidate sex-determining gene appeared feasible. Thus, we proceeded with genome assemblies of each sex.

### 3.2 Genome assemblies

We generated high quality genomes for each sex of *C. guntheri*. Summarized in Table 1, these genomes are on par with or better in quality than other recent cichlid genomes (Tao et al., 2021c). The contig N50 is high, indicating that hifiasm assembled chromosome length contigs for most of the chromosomes (Table 1). The metric for log-scaled probability of error for the consensus base calls, consensus quality (QV), was 65.86 in the male and 65.36 in the female, indicating a very accurate consensus. Additionally, k-mer completeness was 94.35% in the male, and 91.45% in the female. As the region sex-determining region on LG19 is small, and has not accumulated a large number of repeats, it assembled as a single contig in the female. A single join was made of two large contigs in the male LG19, but this breakpoint does not appear to be in the sex-determining region.

**TABLE 1:** Genome assembly statistics.

	*C. guntheri* (XY male)	*C. guntheri* (XX female)
Sequencing type	PacBio HiFi	PacBio HiFi
Genome size	866,651,555	834,188,889
Genome coverage	101.71X	113.189X
Number of chromosomes	24	24
GC%	41.6%	41.7%
Scaffold N50	34,972,419	33,683,495
Contig N50	33,939,210	31,114,759
Complete BUSCOs	98.7%	96.4%
Complete and single-copy BUSCOs	97.5%	95.0%
Complete and duplicated BUSCOs	1.2%	1.4%
Fragmented BUSCOs	0.2%	0.3%
Missing BUSCOs	1.1%	3.3%

*C. guntheri* has 2n = 48 chromosomes (Ozouf-Costaz et al., 2017), and these additional two chromosomes assembled cleanly with clear telomeric regions (Supplementary Figure S2; Supplementary Tables S3–S5). Their homology to other cichlid genomes was confirmed via sequence alignments. LG24 aligned against parts of *O. aureus* LG23, and LG25 against parts of *O. aureus* LG3. LG3 is itself a result of a fusion of two chromosomes, which is thought to have occurred at the base of the African cichlid lineage (Poletto et al., 2010; Conte et al., 2017; Ozouf-Costaz et al., 2017). The LG3 fusion and further fusion with a B chromosome in the Oreochrominii has been previously characterized in cichlids (Conte et al., 2021), so we investigated which chromosome corresponded to each region of the fused LG3. The genes *sarcs2* and *adamsts1* of *O. niloticus* aligned to LG3 from the *C. guntheri* male assembly. The genes *poln*, *nhs12*, *tec*, and *ccdc171* aligned exclusively to LG25. This suggests that the chromosome called LG25 in this assembly corresponds with the LG3a from the Conte et al., 2017 study, and LG3 in our assembly corresponds to *O. niloticus* LG3a’.

Two incorrect joins were made by hifiasm in the female assembly. The first was an incorrect assembly that was broken into LG16 and LG20, respectively using alignments comparing female contigs to the male assembly, and female reads to the female assembly. These were visually inspected in IGV (Thorvaldsdóttir et al., 2013) to determine that the contig produced by hifiasm was not supported by the reads. The same process was repeated for the second contig that broke into parts of LG11 and LG15 that required further manual joining to other contigs to form a more complete scaffold.

Once the assemblies were complete, we mapped the pool-seq reads to the new *C. guntheri* male assembly. The same signal was present on LG19, though it was stronger against the *C. guntheri* reference than against the *O. niloticus* reference (Figure 1B). In the top 1% analysis, LG19 had 9 XY windows and 1 ZW window and the top 100 kb window (340 XY SNPs) was on LG19. The highest non-LG19 window was on LG7 with 86 XY SNPs. LG14 showed significant signal in the Dunn’s test but did not appear in the top 1% windows. The signal on LG3 was weak and diffuse compared to LG19 and had both XY and ZW signal in equal strength. The highest window on LG3 contained 35 XY SNPs. Thus, we conclude that the sex chromosome in *C. guntheri* is LG19.

### 3.3 Structure of the sex determining region

**FIGURE 2:**
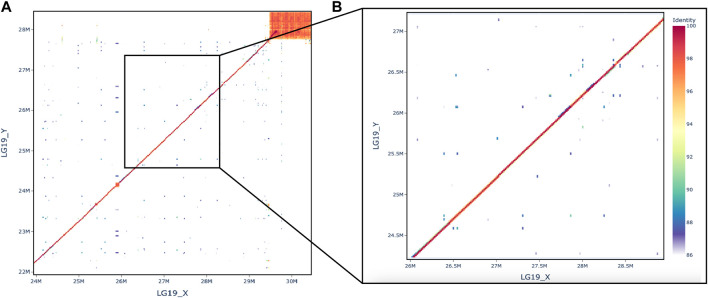
Dotplot comparing X and Y, **(A)** full chromosome and **(B)** the zoom on the region where *rin3* is at 27.6 Mb.

To understand the structure of the sex-determining region, we aligned both the HiFi sequencing reads and the pool-seq reads against the new male genome. Additionally, we aligned the X and Y assemblies to each other. There is no evidence for a structural rearrangement in the sex determining region of LG19 (Figure 2). The percent identity between the X and the Y is high, with the exception of the region at the end of the chromosome. This sub-telomeric region has likely accumulated a large quantity of repetitive DNAs due to the low recombination rate at the ends of cichlid chromosomes (Conte et al., 2021).

In the narrow region of sex differentiation we found the following genes, listed in order from left to right: acireductone dioxygenase 1 (*adi1*), dynein axonemal light chain 1 (*dnal1*), tubulin epsilon and delta complex 1 (*tedc1*), parathyroid hormone 2 (*pth2*), ras and rab interactor 3 (*rin3*), and LOC120434950 which is uncharacterized and had no informative hits when BLASTed against the GenBank database. *Perk2* was initially considered as a candidate gene based on the *O. niloticus* genome, however it became apparent that this gene is misannotated in the *O. niloticus* genome, likely because of the proline-rich domain. Alignments of the sex-determining region against the *O. niloticus perk2* on LG19 were poor (<50% alignment), and the annotation is 7,143 bp with only two exons. When we searched the NCBI genome data viewer, the full copy of *perk2* appears on LG3 and is 13,618 bp long with seven exons. Moving forward, we utilized the *O. aureus* annotation for this region.

While there is a high concentration of male-specific SNPs in this region, it is likely that many of these occur in introns or other non-coding regions. Identifying changes to enhancers and other regulatory regions is challenging without extensive functional annotation, so we chose to focus on SNPs that caused changes in the encoded proteins. Thus, we evaluated SNPs that resulted in a non-conserved amino acid change and were likely to impact the structure or function of the resultant protein. Only two genes from the list, *adi1* and *rin3*, had a non-conserved amino acid change in *C. guntheri* that also differed from the reference cichlid species (Table 2). Any other amino acid changes to genes were conserved or shared with an outgroup species. *Adi1* had a change at G185R, however this change was present in the female sequence (X) but not the male sequence which could result in a functional Y and a non-functional X.

**TABLE 2:** Non-conserved amino acid changes in candidate sex-determining genes found in the region with the highest number of male sex-specific SNPs.

Gene	Different from outgroups but same in *C. guntheri* X and Y	X different from outgroups	Y Different from outgroups
*adi1*	M68T, R128S	G185R	—
*tedc1*	T10M, P13L	—	—
*pth2*	T10M, G116R	—	—
*rin3*	S79F, P126V, R192C, A200V, L300P, S334Del, P407S, V428A, R430S, G538Del, G540K, E544G, E546G, P582S, S587C V644G, L657P, S732P, Q766L, S847A, S881C, A1033S, P1096L, A1120V	N297K E535Del, K536Del, E537Del	P76S, P87S N297E P384S

*Danl1* was not included in this analysis as it was either not well-conserved between the reference species, or the reference proteins were incomplete.

### 3.4 Candidate sex determining gene

**FIGURE 3:**
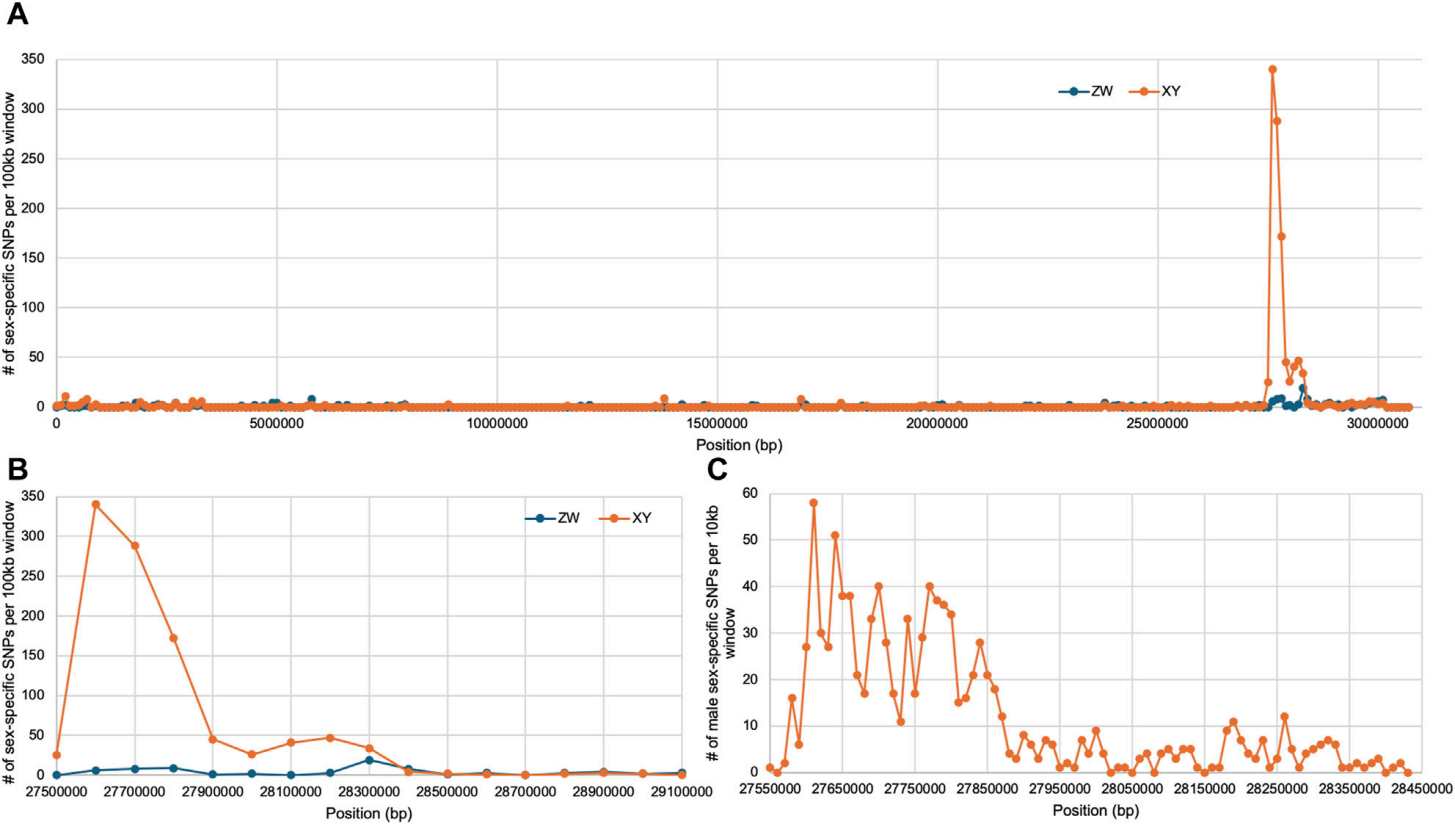
Sex-specific SNP density on LG19, *rin3* is at 27.6 Mb. **(A)** Full chromosome with both XY and ZW SNPs plotted in 100 kb windows, **(B)** zoomed in plot on the sex-determining region in 100 kb windows, **(C)** zoomed in plot on sex-determining region in 10 kb windows.

**FIGURE 4:**
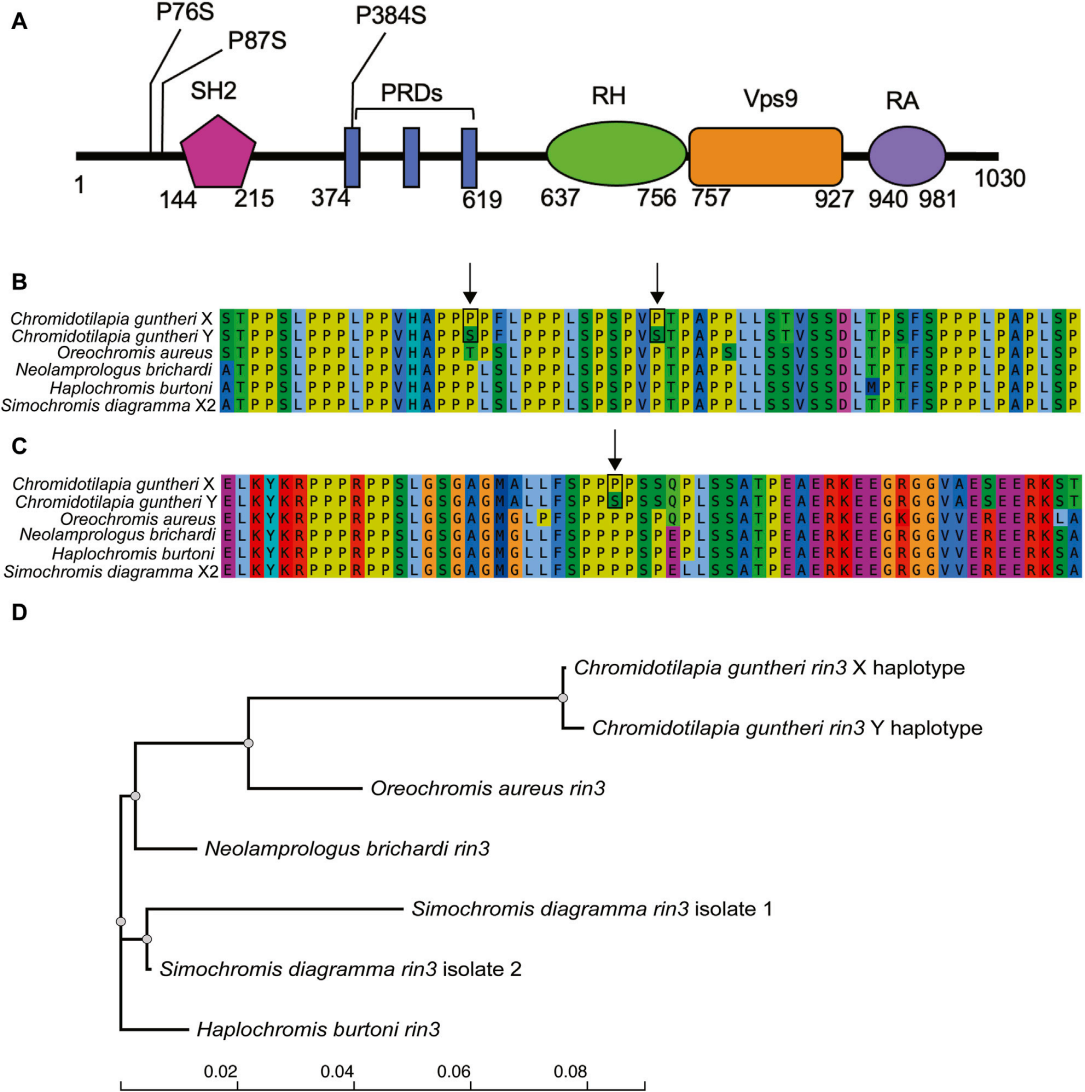
Variation in the candidate gene *rin3*. **(A)** domains of *rin3* with positions of non-conserved amino acid changes (Kajiho et al., 2003), **(B)** alignment of *C. guntheri* and reference cichlid species corresponding to the first two amino acid changes which are indicated with an arrow, **(C)** alignment of *C. guntheri* and reference cichlid species corresponding to the amino acid change in the PR domain, **(D)** gene tree of *rin3* in of *C. guntheri* and reference cichlid species.

We focused our analysis on *rin3*, as it features the highest density of heterozygous SNPs in the male (Figure 3, 27, 607, 824–27,629,697), which included several non-conserved amino acid changes. The multi-sequence alignment showed that the *rin3* sequence on the Y is different from both the reference cichlid species and the X at three amino acid positions (Figure 4). All three of these are non-conserved. Two of these changes, P76S and P87S, occur just before the Src homology 2 (SH2) domain of the protein. The third, P384S, occurs in one of the three proline-rich domains (PRDs). Other changes include position 297, where the X sequence is N297K, and the Y is N297E. This is a conservative substitution, however both E to K are different from the outgroups. A four base pair deletion occurred after position 535 in the X. However, this is part of a repetitive string, and the Y also has a 1 base pair deletion in this region, suggesting that changes to this region have less impact on protein function.

## 4 Discussion

In this study, we used pooled-sequencing of males and females to identify an XY sex-determining locus on linkage group 19 in *C*. *guntheri*. We developed high-quality genome assemblies for a male and female of this species and used these assemblies to characterize a novel candidate gene for sex determination.

### 4.1 High quality genomes for studying sex chromosomes

Our assemblies of *C. guntheri* represent the first chromosome-level assemblies of a Chromidotilapiine cichlid. As *C. guntheri* is an outgroup to the hyper-diverse haplotilapiine cichlids, this is an important resource for understanding African cichlid genome evolution. These genomes assembled with a high N50 and minimal need for scaffolding, resulting in two highly contiguous reference assemblies that allow us to characterize any structural differences between the male and the female genomes. Hi-C might be the gold standard for scaffolding sex chromosomes (Carey et al., 2022), but for the purposes of generating the many reference genomes required for understanding cichlid evolution, our methodology assembling long high-coverage PacBio HiFi reads works well and is more affordable. As shown in Figure 1, the evolutionary distance to the reference sequence has a clear impact on sex chromosome detection. The signal identified on the *C. guntheri* reference is cleaner and stronger than in comparisons using the *O. niloticus* reference. The small sex-determining region means that our analyses were not affected by the usual challenges in phasing polymorphisms across the sex-determining region.

### 4.2 Lack of structural rearrangements

Many sex chromosomes are differentiated by one or more structural rearrangements that reduce recombination between the sex determiner and nearby genes (Jay et al., 2022; Charlesworth, 2023; Olito and Abbott, 2023). However, there is no evidence for an inversion or other structural rearrangement between the X and the Y in *C. guntheri*, and gene order in this region appears to be conserved in cichlids. The region of differentiation between the sex chromosomes is very narrow. This might suggest that the LG19 system is very young, however we do not have information on the sex chromosomes of any closely related species which would help date the origin of this sex chromosome system. It is likely that differentiation is maintained across this small region because of its position in near the end of the chromosome, which in cichlids usually has a low rate of recombination (Conte et al., 2021). The asymmetry of the peak on LG19 is consistent with the proximity of rin3 to the proximity of *rin3* the region of low recombination near the telomere.

### 4.3 Rin3 as the candidate sex determining gene

Of the possible candidate genes in the narrow region on LG19, Ras and Rab Interactor 3 (*rin3*) stands out because it has the highest density of sex-specific substitutions of any genes in the region. The function of *rin3* has been studied with respect to Alzheimer’s disease in humans, however the protein structure is not fully defined (Shen et al., 2022). The known domains include the Src homology 2 (SH2), proline rich regions (PR), RIN homology (RH), vacuolar protein sorting 9 (VPS9/GEF), and the Ub-like Ras association (RA) domain (Kajiho et al., 2003). *Rin3* is distinguished from the *rin1* and *rin2* genes by featuring 3 PR domains (Kajiho et al., 2003). It is involved in the early stages of endocytosis via activation of Rab5 and Rab31, which bind to the VPS9 domain of the protein (Kajiho et al., 2003; Kajiho et al., 2011; Shen et al., 2022). *Rin3* also activates Ras, which is involved in signaling pathways that regulate cellular function (Shen et al., 2022). The SH2 domain is involved in organizing cellular signaling network via protein tyrosine kinases (PTKs) (Pawson et al., 2001; Schlessinger and Lemmon, 2003). The PR domain of *rin3* has been found to bind bridging integrator 1 (*bin1*) (Kajiho et al., 2003), bridging integrator 2 (*bin2*) (Janson et al., 2012), and CD2-associated protein (*cd2ap*) in humans (Rouka et al., 2015; Shen et al., 2020).

Interactions between the domains of *rin3* make it somewhat difficult to discern how changes to a single domain might affect function. Previous work has found that if the SH2 domain is rendered inactive, *rin3* had reduced colocalization with Rab5 indicating that the SH2 domain may self-inhibit the guanine-nucleotide exchange factor (GEF) activity of Rin proteins that are required for interaction with Rab5 (Yoshikawa et al., 2008). Other work has indicated that binding of the SH2 domain to the phosphotyrosine residue from the Ras/MAP signaling pathway inhibits Rab5 activity, while increased *rin3* activity can result in an increase of Rab5 activation and formation of a complex of *rin3-bin1-cd2ap* in early endosomes that interrupts endocytic trafficking (Meshref et al., 2023). While the two amino acid changes fall just before the SH2 domain, cichlids seem to have a larger region before the SH2 domain than humans, which might induce conformational changes that impact binding.

Without functional data, we can only speculate on what effect the changes to the expression or structure of *rin3* might have on the protein and the sex-determination network in *C. guntheri* males. Here, we present two possible mechanisms for how this might occur. The first hypothesis focuses on the role Rab5 plays in endocytosis via both the internalization of the clathrin-coated pits that lead to early endosomes and *via* the caveolin-mediated pathway (Shikanai et al., 2023). Both of these pathways are known to be involved in the endocytosis of TGFß receptors (Le Roy and Wrana, 2005; Chen, 2009; Heldin and Moustakas, 2016). Changes to Rab5 binding might impact the turnover and degradation of TGFß receptors, which has been shown for Rin1 (Hu et al., 2008). Thus, reduced ability of Rin3 to bind Rab5 may impact the TGFß pathway by reducing endocytic activity of these receptors, leaving more TGFß receptors available on the cell surface. An increased number of available receptors might be sufficient to tip the developmental pathway toward maleness. It is also important to note that *bin2*, which binds to Rin3 and plays a role in endocytosis via promotion of membrane bending (Janson et al., 2012), was found as a candidate gene for sex determination in the cichlid tribe Tropheini from Lake Tanganyika (Behrens et al., 2024a). A second hypothesis is less specific to TGFß receptors, but still relates to the role of *rin3* in endocytosis. Endocytosis has been proposed to play a role in sex steroid regulation by enabling sex steroids to enter the cell, in contrast with previous theories that suggested sex steroids freely diffuse through the cell membrane (Hammes et al., 2005; Marko et al., 2022). Thus, reducing the function of *rin3* in the endocytic pathway of males could affect female sex steroid uptake, also tipping the balance toward maleness. Evaluation of these hypotheses will require functional experiments, beginning with the development of CRISPR knockouts of *rin3*.

### 4.4 What genes can become sex-determining

Recent reviews have highlighted three major classes of genes that have become sex determiners in various vertebrate lineages (Curzon et al., 2023; Kitano et al., 2024). In fishes, genes in the TGFß pathway are the most frequently recruited to control sex determination. In the studies published to date, it is the genes for ligands (amh, gsdf, gdf6) or cell membrane receptors (amhr2, bmpr1) that are most often utilized (Pan et al., 2021; Yu et al., 2024). However, these signaling pathways extend deep within the cell, and it is possible that modification of other elements in the pathway might also become Mendelian factors in sex determination. A recent report proposed *id2b* as a candidate gene for sex determination in the arapaima (Adolfi et al., 2021). *Id2* genes are downstream effectors of TGFß signaling, with effects primarily in the nucleus. Here we have proposed *rin3* as a candidate gene for sex determination in *C. guntheri*. *Rin3* may be a modifier of TGFß pathway function by modulating receptor turnover. These results suggest we may have to reconsider the hypothesis that sex determiners typically evolve at the top of biochemical pathways or gene regulatory networks (Wilkins, 1995).

## 5 Conclusion

This work furthers both our understanding of sex-determination in fish, as well as our understanding of the regulatory networks in which sex-determining genes function. Often, the differentiated regions of sex chromosomes span several megabases encompassing dozens of genes. Here we identified a narrow sex-determining region on LG19 in *C. guntheri*, which allowed us to identify *rin3* as a strong candidate for the Mendelian factor regulating sex determination in this species. We also developed high quality male and female genome assemblies for *C. guntheri*, an outgroup to the extraordinary radiation of cichlids in East Africa, which will be an important resource for evolutionary studies of this group.

## Data Availability

The datasets presented in this study can be found in online repositories. The names of the repository/repositories and accession number(s) can be found below: https://www.ncbi.nlm.nih.gov/, JBDKXC000000000; https://www.ncbi.nlm.nih.gov/, JBDKXD000000000; https://www.ncbi.nlm.nih.gov/, PRJNA1100981.
