# Supplementary material for: Genome assemblies for Chromidotilapia guntheri (Teleostei: Cichlidae) identify a novel candidate gene for vertebrate sex determination, RIN3
Source: Front Genet. 2024 Aug 16;15:1447628. doi: 10.3389/fgene.2024.1447628 (PMC11361979; doi:10.3389/fgene.2024.1447628)
Supplement: Supplementary file 2 [file Table1.DOCX]

Supplementary Material

**Supplemental Figures**

**
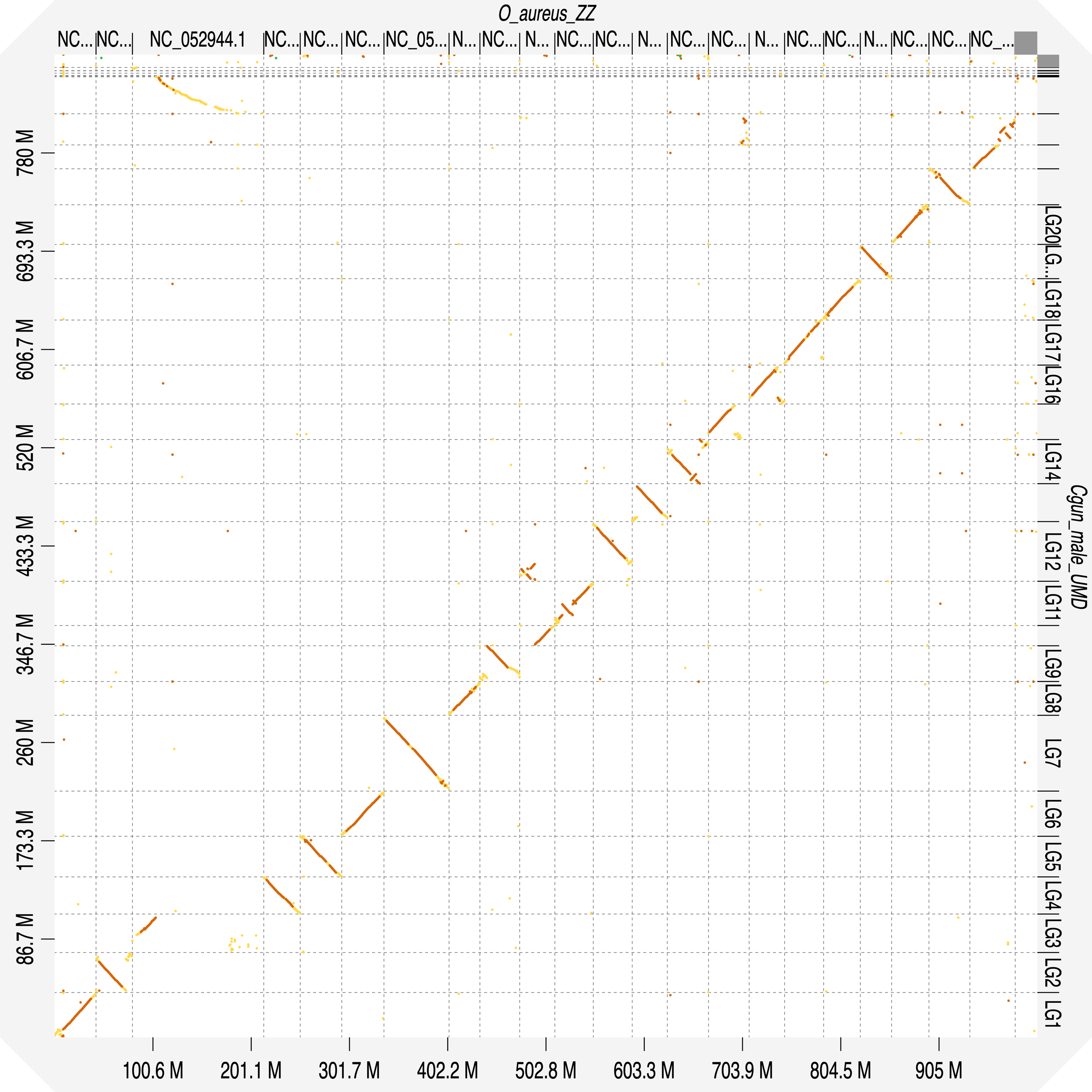
**

Supplementary Figure 1. Dot plot of the *C. guntheri* male genome assembly against the *O. aureus* genome assembly.

a


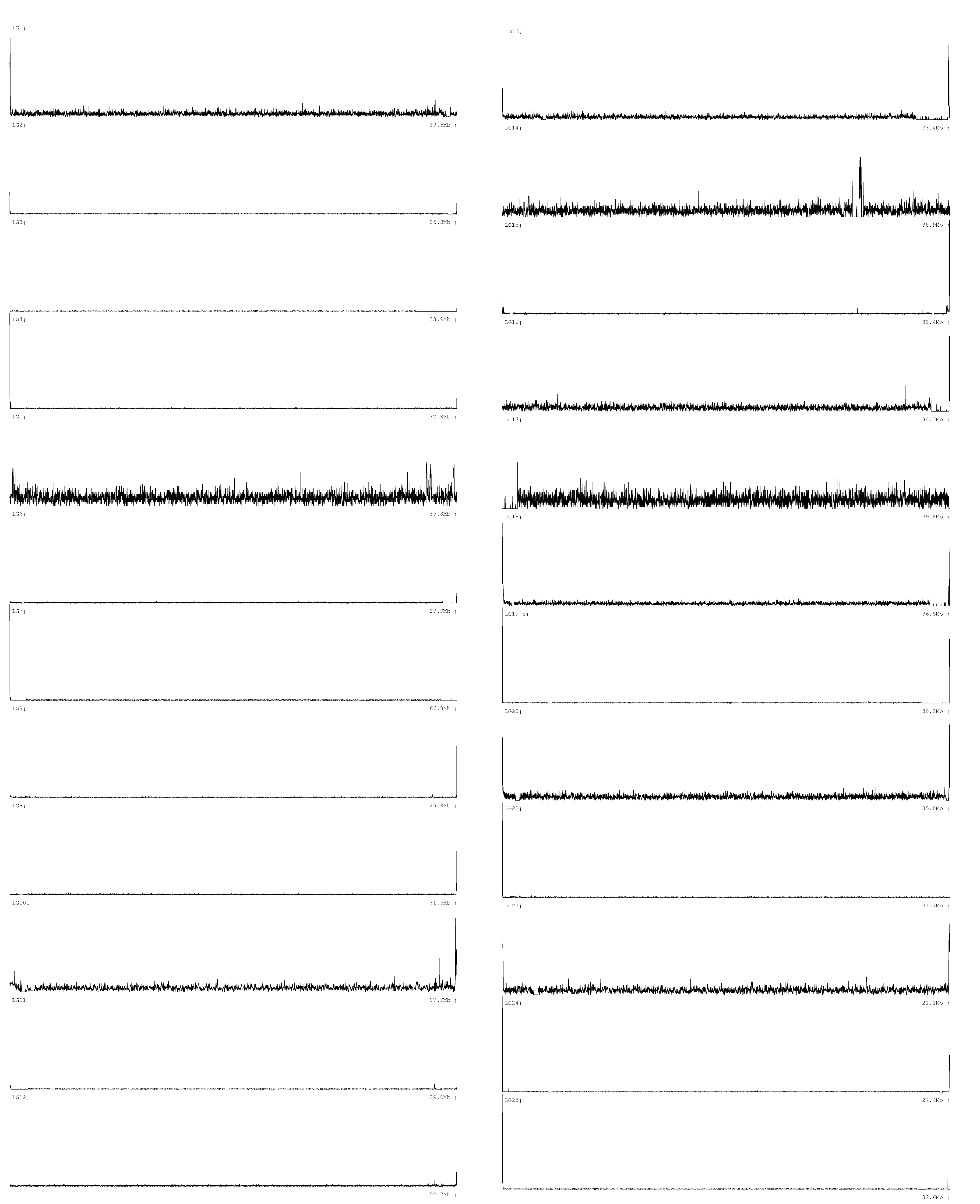


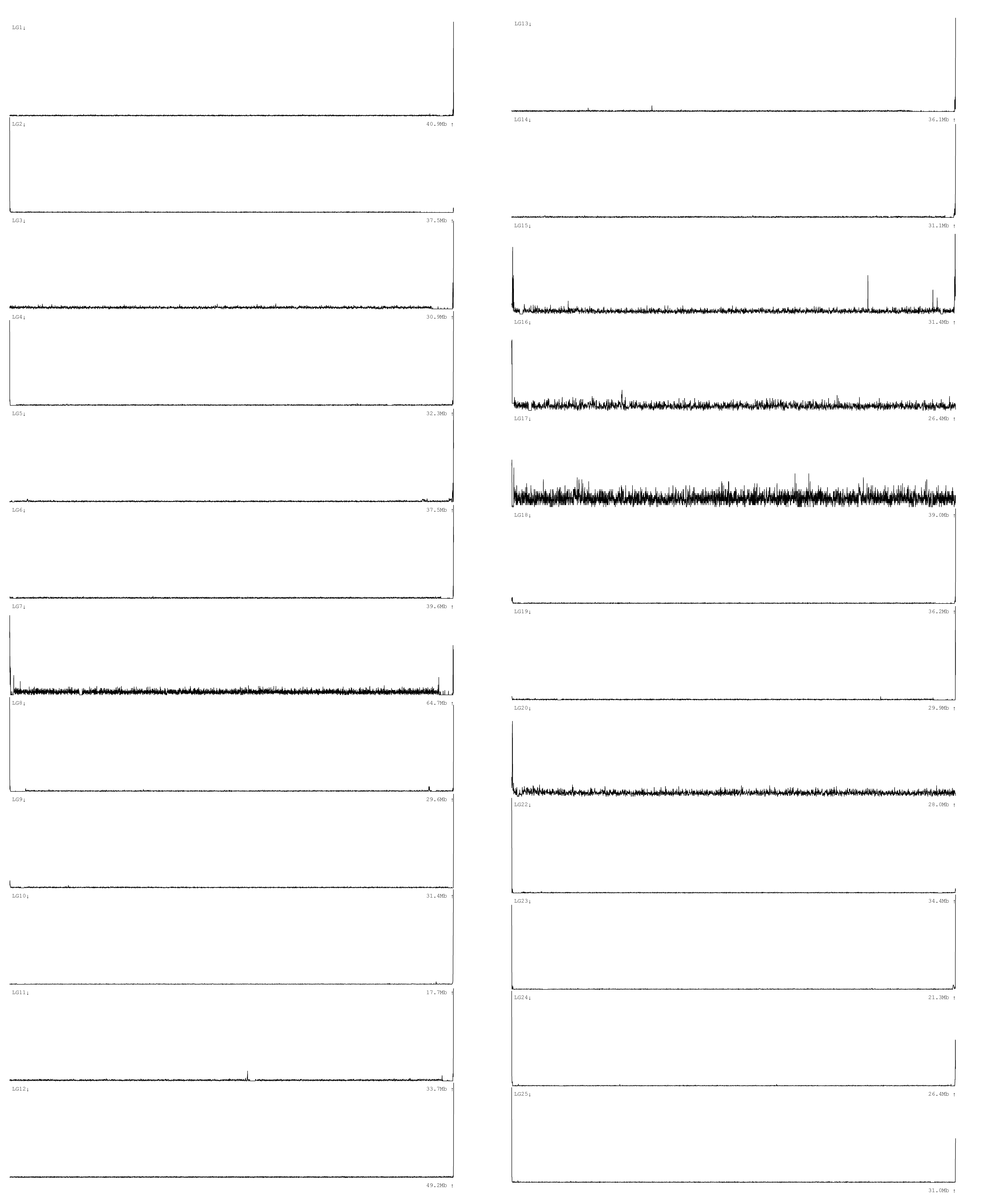


b

Supplemental Figure 2. Plots of the distribution of the telomeric repeat AACCCT for each chromosome a) male assembly, b) female assembly. Chromosomes with a tall peak at each end represent telomere-to-telomere assemblies. Chromosomes with one peak represent assembly of only one telomere. Chromosomes with no peak and a lot of noise indicate failure to assemble either telomere.
